# Stability of crushed tedizolid phosphate tablets for nasogastric tube administration

**DOI:** 10.1186/cc14194

**Published:** 2015-03-16

**Authors:** G Kennedy, J Osborn, S Flanagan, N Alsayed, S Bertolami

**Affiliations:** 1 Cubist Pharmaceuticals, Lexington, MA, USA; 2 Cubist Pharmaceuticals, San Diego, CA, USA; 3 Cubist Pharmaceuticals, Zurich, Switzerland

## Introduction

Tedizolid phosphate, a novel oxazolidinone antibacterial prodrug recently approved by the US Food and Drug Administration for the treatment of acute bacterial skin and skin structure infections, is available as oral (that is, tablets) and intravenous formulations. The clinical pharmacokinetics of tedizolid, the active moiety of tedizolid phosphate, are similar when orally administered tedizolid phosphate is given as powder in a capsule or as tablets. This suggests that crushing tablets prior to administration is unlikely to alter tedizolid pharmacokinetics, provided no drug is lost during administration. To determine whether the expected dose of tedizolid phosphate can be delivered via nasogastric (NG) tube in critically ill patients who have difficulty swallowing, this study evaluated the stability and recovery of tedizolid phosphate 200 mg tablets after crushing, dispersion in water, and passage through an NG tube.

## Methods

For each assay, run in triplicate, one 200 mg tablet of tedizolid phosphate was crushed, dispersed in water, drained under gravity through one of two types of NG tubes (type 1, Kangaroo Nasogastric Feeding Tube, 10 Fr 43" (109 cm); type 2, Salem Sump Dual Lumen Stomach Tube, 18 Fr/CH (6.0 m) 48" (122 cm)), and collected for recovery analysis by high-performance liquid chromatography with UV detection. To analyze the chemical stability of the crushed tablet dispersed in water, the aqueous preparation was assayed initially after dispersion and again after 4 hours at room temperature, without NG tube passage. The prespecified limit for tedizolid phosphate in recovery samples was 90 to 110% of the dose. Limits were also specified for levels of certain impurities.

## Results

The average and individual recovery values of tedizolid phosphate were within 90 to 110% of the 200 mg dose when crushed tablets, dispersed in water at room temperature, were transferred through the 2 NG tubes (type 1: 95.8%; type 2: 93.6%). There was no significant change in recovery values after 4 hours of storage at room temperature (93.9% initially and 94.7% after 4 hours). Results for degradation products and impurities were also within specified limits in NG recovery samples and in the 0-hour and 4-hour aqueous preparations.

## Conclusion

The stability and recovery of tedizolid phosphate were not influenced by crushing the tablets and passing through an NG tube. Therefore, administration of crushed tedizolid phosphate tablets to patients is unlikely to alter the pharmacokinetics of tedizolid compared with whole tablets.

